# Endophytic Fungi in Species of *Artemisia*

**DOI:** 10.3390/jof4020053

**Published:** 2018-05-01

**Authors:** Andreea Cosoveanu, Raimundo Cabrera

**Affiliations:** Facultad de Ciencias—Sección Biología, Dept. Botanica, Ecologia & Fisiologia Vegetal, Universidad de La Laguna, Apdo. 456, 38200 La Laguna, Spain; rcabrera@ull.edu.es

**Keywords:** medicinal plants microbiome, phylogeny, bioactivity, rare fungal species

## Abstract

The genus *Artemisia*, a collection of ~400 hardy herbaceous plant and shrub species, is an important resource contributing to chemistry, medicine, agriculture, industry, and ecology. Its communities of endophytic fungi have only recently begun to be explored. Summarized from studies conducted on the fungal endophytes in *Artemisia* species, both fungal phylogenetic diversity and the associated bioactivity was examined. Isolations from 14 species of *Artemisia* have led to 51 genera of fungal endophytes, 28 families, and 18 orders. Endophytes belonged mainly to *Ascomycota*, except for two taxa of *Cantharellales* and *Sporidiobolales*, one taxon of *Mucoromycota*, and one species of *Oomycota*. The mostly common families were *Pleosporaceae*, *Trichocomaceae*, *Leptosphaeriaceae*, and *Botryosphaeriaceae* (relative abundance = 14.89, 8.51, 7.14 and 6.38, respectively). In the search for bioactive metabolites, 27 novel compounds were characterized and 22 metabolites were isolated between 2006 and 2017. The first study on endophytic fungi isolated from species of *Artemisia* was published but 18 years ago. This summary of recently acquired data illustrates the considerable diversity of biological purposes addressed by fungal endophytes of *Artemisia* spp.

## 1. State of the Art

*Artemisia* is a genus of plants highly valued as a source of metabolites useful in, for example, medicine and biopesticides. Phytochemical analyses showed the main compounds in *A. vulgaris* to be flavones, flavone glycosides, flavanones, flavonols, flavonol glycosides, and volatile compounds such as α-pinene, camphor, camphene, germacrene D, 1,8-cineole, β-caryophyllene, α-thujone, 1,8-cineole, sabinene, β-thujone, β-caryophyllene oxide, neryl 2-methylbutanoate, β-eudesmol, and bornyl 3-methylbutanoate [1,2,3,4]. A review on the chemistry of 15 species of *Artemisia* resulted in 839 compounds with mainly terpenoids, flavonoids, coumarins, caffeoylquinic acids, sterols, and acetylens [5]. Biological activity was detected in a series of compounds such as eupatilin (anticancer) [5]; artemisolide (anti-inflammatory) [6]; α-thujone (toxicity against adult mites) [7]; chamazulene, 1,8-cineole, and β-caryophyllene (toxicity against the cigarette beetle) [8]; luteolin (anti-inflammatory, anticancer, antimicrobial, antioxidant) [9]; and eriodictyol (anti-inflammatory properties, beneficial effect in treatment of diabetic retinopathy, emmenagogue) [10,11,12]. Also, *Artemisia annua* remains the main commercial source for artemisinin [13]. Nevertheless, essential oils; organic fractions of hydrolate byproducts; and extracts of various species of *Artemisia*, wild and domesticated (*A. absinthium*, ^®^Cvar. Candial, Spain), proved to have potential in further commercial use with effects such as anti-toxoplasmosis [14], anti-mosquitoes [15], nematicidal [16,17], larvicidal against *Pieris brassicae* [18] and reduction of the longevity and fecundity of *Tetranychus urticae* [19]. Extensive reviews describe (i) the conservation status, phytochemistry and biological activities of the *Artemisia* genus in the Iberian Peninsula and two Macaronesian archipelagos [20]; (ii) the chemical composition and biological activity of essential oils in various species of *Artemisia* [6] and (iii) the phytochemistry and pharmacological and biotechnological potential of *A. vulgaris* [4].

**Figure 1:**
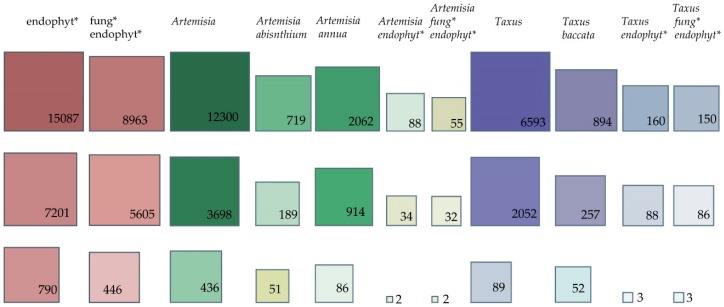
Survey of the literature in databases (Scopus—first upper line of squares; PubMed—middle line with squares; and CAB Direct—bottom line with squares) with indexed keywords related to host plants and endophytes. * = Keywords were used as shown in the figure except for the PubMed database, where the use of wildcards is irrelevant in a standard search as it uses only the root of the term for the generating hits. The search was performed for the title, abstract and keywords or descriptors, where possible (i.e., in Scopus and CAB Direct). Values in squares are the number of publications. Squares are proportional to the number of publications. Interpretation of squares scaling was calculated per group of data as follows: cluster 1—endophytes and fungal endophytes, cluster 2—*Artemisia* and related keywords, cluster 3—*Taxus* and related keywords; the highest number of publications per each cluster was considered 100%. Transformation to log_10_ was performed to reduce the skewness and facilitate interpretation of the squares.

A survey of the literature shows that this genus has engaged many researchers, with 12,300 publications in the Scopus library, almost double that of the genus *Taxus* (Scopus library—6593 entries). Although *Taxus* is associated with US$1B per annum worth of sales of the cytostatic drug taxol, the fact that *Artemisia* is a medicinal plant genus with 474 accepted species [21] and multiple uses may explain its relative dominance of the literature. The present survey also reveals the dominance of the plant-focused research rather than that devoted to the plant–endophyte associations. As may be observed in Figure 1, whether we consider one or all databases, the numbers show that up to 0.9% of the number of the publications containing “*Artemisia*” as a keyword include “endophyte” as a keyword (Scopus—88 entries, 0.7%; PubMed—34 entries, 0.9%; and CAB Direct—two entries, 0.45%). For *Taxus* and endophyte, the comparative number of publications was up to 4.28% (Scopus—160 entries, 2.42%; PubMed—88 entries, 4.28%; and CAB Direct—three entries, 3.37%).

Comparing the keywords “*Artemisia* endophytes” and “*Artemisia* fungal endophytes” in Scopus, the number of publications containing “*Artemisia* fungal endophytes” represents 62.5% of the number of publications containing “*Artemisia* endophytes”, except in CAB Direct and PubMed where no relevant difference was observed. We might extrapolate that the rest of 37.5% of the publications regard bacterial endophytic communities in *Artemisia*. Similar numbers are shown for the comparison within the *Taxus* genus.

*Artemisia annua* was highly rated with three times more publications than for *Artemisia absinthium* (Scopus library—2062 hits versus 719, respectively). These values reflect the great interest in artemisinin, produced by *Artemisia annua*, which is an important drug used to treat malaria and cancer. Various fungi have been investigated as elicitors or direct producers of artemisinin. Up-to-date information in Table 2 summarises the findings from research into endophytes of *Artemisia* endophytic communities.

## 2. *Artemisia* Fungal Endophyte Identification and Diversity

Overall, the identification of the fungal endophytes in *Artemisia* spp. is made based on morphological characterization and molecular analysis using (i) 28S ribosomal DNA spanning domains D1 and D2 and (ii) nuclear ribosomal DNA sequences, including both the internal transcribed spacers (ITS1 and ITS2) and the 5.8S gene region. Three studies investigated the phylogenetic analysis of the *Artemisia* spp. fungal endophytes [22,23,24]. Although the number of studies is small, interesting facts are brought to light in terms of diversity and plant colonization. For *Artemisia annua*, the endophytic fungal infection frequency was slightly higher in the roots of cultivated plants (20.9%) than in the roots of wild plants (16.7%) [25]. Moreover, the authors showed that the latter roots harboured more rich fungal taxa, which supports the hypothesis that wild plant species are predisposed to be hosts for rich and novel mycoflora [26,27]. The differences can be extended to fungal pathogens, where 55% of the fungal communities in wild fruits were not present in their homologous cultivated species [28]

Strains of *Aplosporella prunicola*, *Chaetomium* sp., *Macrophomina phaseolina*, *Nectria mauritiicola*, *Neofusicoccum australe*, *Pestalotiopsis* sp., and *Stachybotrys longispora* were isolated only from one nutrient medium and only from *Artemisia thuscula* collected in Tenerife island, in a study comparing La Palma and Tenerife in the Canary Islands [24]. Diversity indices showed that plant individuals collected in La Palma had higher diversity values in terms of species richness, diversity of taxa, abundance of rare species, and species evenness. The study indicates the relevance of the exploration of different plant tissues and usage of wider number of growth media to obtain diversity. Comparing arbuscular mycorrhizal fungi (AMF) with dark septate endophytes (DSE) and other types of endophytic fungi (EF), a low colonization frequency of AMF was detected in roots, while EF were more frequently found in *Artemisia annua* [25].

Endophytic fungi were isolated from *Artemisia argyi*, and *Pleosporales* was found to be the most represented group, with three species of *Alternaria* present [29]. It is worth mentioning that Qian et al. [29] reported the presence of *Rhodotorula* sp. and *Fusarium* sp. in *Artemisia argyi*. Myrchiang et al. [30] investigated the endophytic fungi associated with *Artemisia nilagirica*, and isolated among the dominant clade of *Ascomycota*, one strain of *Pythium intermedium* (Oomycota) and one strain of *Rhizopus oryzae* (Zygomycota). Comparing the colonization of three plant parts (i.e., root, stem, and leaf), the authors found that the highest diversity was in the roots (i.e., 14 species), followed by stems (i.e., 10 species), and least in the leaves (i.e., six species). Similarly, in *Artemisia thuscula* were isolated 29 distinct morphotypes: 20 from roots, seven from stems, and two from leaves [31]. In addition, Myrchiang et al. [30] observed that from all fungal endophytic species, only *Phoma eupyrena* was found to be most dominant in all plant samples; the other species having a certain preference for one or a maximum of two plant parts. Huang et al. [22] classified 108 fungal isolates obtained from three medicinal plant species, *Artemisia capillaris*, *Artemisia indica*, and *Artemisia lactiflora*, using morphological identification. In total, 42 isolates were obtained from the host *Artemisia capillaris* (five isolates from leaves, 15 isolates from stems, and 22 isolates from inflorescences). In total, 39 strains were isolated from the host *Artemisia indica* (18 isolates from leaves and 21 from stems). Twenty-seven fungal strains were isolated from the host *Artemisia lactiflora* (16 isolates from leaves and 11 from stems). Among them, *Alternaria*, *Colletotrichum*, and *Phomopsis* were common, especially *Alternaria* spp. (25% relative isolation frequency) and *Colletotrichum* spp. (20.4% relative isolation frequency). One strain was isolated from the stems of *Artemisia capillaris* and was similar to *Drechslera* sp. Among the three plant hosts, the highest endophytic colonization rate occurred in *Artemisia capillaris,* which had the highest fungal diversity. Five fungal isolates belonging to *Aureobasidium pullulans, Ephelis*, *Pestalotiopsis*, and *Pleosporaceae* were only obtained from *Artemisia capillaris. Xylaria* species appeared to be the most common endophytic fungi in *Artemisia indica.*

Seven *Artemisia* species were sampled in two locations (Qichun and Wuhan), and 21 fungal endophytic species were isolated from stems and identified as *Diaporthe*, *Colletotrichum*, *Nigrospora*, *Botryosphaeria*, *Aspergillus*, *Penicillium*, *Neofusicoccum*, *Cercospora*, *Rhizoctonia*, *Alternaria*, and *Curvularia* [23]. The highest incidences of colonization frequency per plant were attributed to *Nigrospora sphaerica* in *Artemisia* sp., *Nigrospora oryzae* in *Artemisia argyi*, *Alternaria alternata* in *Artemisia subulata* and *Artemisia tangutica*, and *Botryosphaeria dothidea* in *Artemisia lavandulifolia*. This was the first report of *Nigrospora*, *Neofusicoccum* and *Curvularia* species in *Artemisia* spp. As for plant species specificity, only *Nigrospora sphaerica, Nigrospora oryzae* and *Alternaria alternata* were present in various plants; except for *Cochliobolus geniculatus*, isolated from *Artemisia brachyloba* and *Artemisia argyi.* Thus, few endophytic fungi were found to be entirely restricted to particular plant species, but significant differences were found in the frequency of colonization of individual morphotaxa. The study highlighted the *Alternaria* genus as the dominant one, having the reputation of being one of the cosmopolitan endophytes reported. *Nigrospora* displayed the second highest rate of occurrence in this analysis, followed by *Botryosphaeria* whose incidence has been described more in woody plants.

A heat map table (Table 1) describes the relative abundance of genera, families and orders of the endophytic mycopopulation of *Artemisia* species. The most isolated taxa belong to *Pleosporales*, *Hypocreales*, *Xylariales*, *Botryosphaeriales*, and *Eurotiales* (relative abundance % = 25, 17.31, 9.62, 7.69, and 7.69, respectively). Yet these numbers do not reflect the diversity of the families included, as *Pleosporales* and *Hypocreales* have been found each with taxa belonging to four families while *Hypocreales* and *Xylariales* were found with four and two families, respectively. *Phomopsis*, *Alternaria* and *Penicillium* were the most isolated genera (relative abundance % = 36.36, 36.36 and 33.33, respectively). While many of the genera (Table 1) are frequently found as endophytic strains in medicinal plants [32,33,34,35], temperate grasses [36], tropical and subtropical rainforests [37] and also as fungi associated with *Vitis vinifera* [38], some are quite rare: *Aplosporella*, *Ramichloridium*, *Ephelis*, *Stachybotrys*, *Drechslera*, *Biscogniauxia*, *Edenia* and *Thielavia.*

**Figure 2:**
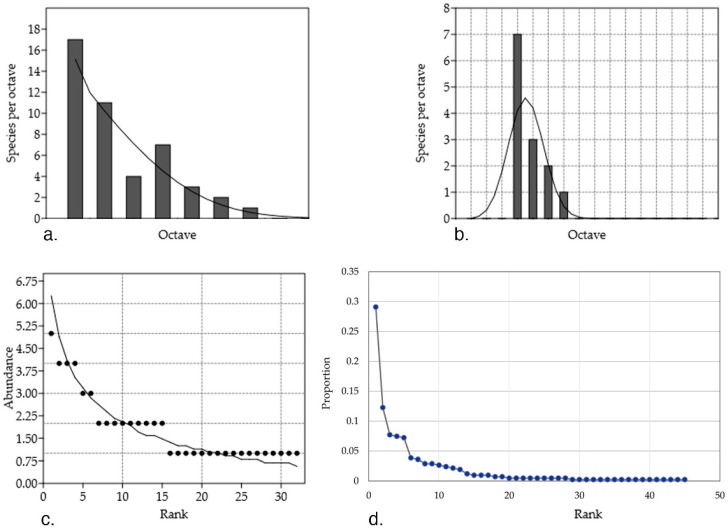
(**a**) The number of all endophytic fungi (EF) genera plotted in a log-normal distribution (fitted mean = 0.05, variance = 0.68, chi^2^ = 2.81, *p* = 0.42) for different abundance octaves. The octaves refer to power-of-two abundance classes. The species which theoretically are expected to be present are veiled, as their low abundance prevents them for being represented in the sample. The log-normal distribution was a poor fit to this data due to an overabundance of rare species compared with that expected from the log-normal curve; (**b**) Abundant EF genera (> eight individuals) predicted by a log-normal distribution (mean = 1.28, variance = 0.12, chi^2^ = 3.9, *p* = 0.04); a poor fit, as the first interval is dominated by a genus, namely *Alternaria*; (**c**) Rare EF species (< eight individuals) predicted by a log series distribution (α = 29.32, x = 0.66, chi^2^ = 0.72, *p* = 0.99), where many rare genera (17 genera with one strain) are represented by a jagged line because predicted values are rounded to the nearest number; (**d**) Rank abundance curve of the endophytic fungal genera. The slope is extremely steep at the beginning at the curve, meaning that evenness of the community is low, where few of the genera are highly abundant. For (**a**–**c**), only absolute counts, as opposed to relative proportions, were considered, while for (**d**), relative proportions were considered. Abundance model were calculated with PAST 3.18 software (Øyvind Hammer, Natural History Museum, University of Oslo, Norway) [50].

Rare and widely abundant species inhabiting *Artemisia* host plants are different from each other in distribution–abundance relationship (Figure 2). Some of the considered shortcomings of the study are: (i) data is based on purely culture-dependent strains; (ii) the sampling method differs among studies (for instance, in terms of considering two different strains of the same species, there is no standardized method or at least an extended discussion in the literature); and (iii) the lack of information hidden behind the studies which are interested in showing the bioactivity of certain species (or else) without mention on the abundance of strains.

The plotted data shows that the theoretical assumption of a log-normal distribution of the abundance in nature, that is, endophytic fungal communities, where few common taxa have a high abundance and also few taxa are scarcely found, does not fit to the collected data; to name relevant examples: (i) abundant genera (between three and 11 mentions) *Alternaria*, *Nigrospora*, *Penicillium*, *Phomopsis*, and *Neofusicoccum* (highest RA % = 75, 75, 36.4, 33.3, 41.7 and 30, respectively); (ii) least abundant (between two and seven mentions) *Pestalotiopsis*, *Colletotrichum*, *Diaporthe*, *Acremonium*, *Preussia*, and *Chaetomium* (highest RA % = 11.8, 19.8, 16.7, 13, 11 and 11.8, respectively); and (iii) singleton (with one mention) taxa such as *Edenia*, *Drechslera*, *Stachybotrys*, and *Ephelis* (RA % = 2.7, 0.9, 1.2 and 1.8, respectively). Studies show that the extension of data sampling is directly proportional to the unveiling of the missed taxa [44]. So, the deviations from the log-normal distribution can be used to estimate the inventory size and assume that more rare species would be identified or fall into common categories had there been more sampling and computation.

In this data collection, 71% of the species were less abundant (between one and eight mentions of strains) with 37.77% of the species being singleton. The cosmopolitan theory has been previously argued and demonstrations against its postulates (i.e., everything is everywhere) have shown patterns of biogeography for the rare marine microbial biosphere [45]. Furthermore, 59% of the endophytic fungal species found in tropical forest of Panama were singletons [46] while incidences of endophytic colonization depend on environmental and geographical factors [33]. Isolated ecosystems are known for their specific biodiversity. In this dataset, 28% of the taxa were isolated from *A. thuscula* in the Canary Islands while the rest of the genera were found among all regions (China, Hong Kong, India, La Palma, and Tenerife).

Studies on endophytic communities isolated from a plant genus host are encouraged, as besides the implications of the ecosystem, the relations with the host are considered to be specific (chemical, biochemical and physiological levels as well as the biological life cycle). Furthermore, studies have shown a presumable specificity or preference of various endophytic taxa for their hosts [47,48,49].

This difference in the distribution–abundance relationship between the rare and cosmopolitan taxa raises a question: what determines the exclusive occurrence of singletons; is it sampling size, host, or site?

## 3. Biological Activity of *Artemisia* Endophytes

Extremely diverse biological activity demonstrated by endophytic fungi associated with *Artemisia* spp. has been a driving force in the search for active endophytic strains in *Artemisia annua* [41,51,52,53,54]. Two new compounds have been discovered, namely leptosphaerone [43] and leptosphaeric acid [55] which were obtained from *Leptosphaeria* sp. (Table 2). Fungal endophytes in this plant species were mainly evaluated for their elicitor role in artemisinin production, for their direct production of the molecule or as their role in planta enhancement of artemisinin. Likewise, Lu et al. [40] isolated *Colletotrichum* sp., an endophytic fungus producing-the plant hormone indole-3-acetic acid (IAA). Plant growth-promoting traits for example increase in plant biomass and chlorophyll content in leaves was demonstrated using a dual symbiotic culture of *Piriformospora indica* and *Azotobacter chroococcum* [56]. In addition, a positive influence of *P. indica* filtrate on overall growth and development of micro—propagated plants was observed [57]. Chlorophyll a, chlorophyll b, and carotenoid content were found at the highest rates in *Piriformospora indica*—associated plants compared to the control and autoclaved *Piriformospora indica* treated plants [58]. Substantial increase in height and leaf area of *Artemisia annua* was observed in plants treated with fungal elicitor extracts obtained from *Cochliobolus lunatus*, *Colletotrichum gloeosporioides*, *Acremonium persicinum* and *Curvularia pallescens* [59].

Monitoring the developmental effects of the *P. indica* endophytic fungus on its *Artemisia annua* host plant, researchers studied the effect on artemisinin production [56,57]. This was proved not only to be an antimalarial drug for human health, but also known to be very effective against a wide spectrum of microorganisms including protozoa, bacteria, fungi and viruses as well as serving as a selective insecticide and phytoalexin [60] and also being used in cancer treatment [61]. In addition, oligo- or polysaccharides derived from endophytic *Colletotrichum* sp. were used successfully as elicitors to stimulate artemisinin production in *Artemisia annua* hairy root cultures [53,54,62]. *Artemisia annua* plants resulted in a 3.47-times increase (*p* < 0.01) in artemisinin production after being treated with *Curvularia pallescens* fungal elicitor extract [59]. Also, mycelium extracts of *Penicillium oxalicum* promoted artemisinin biosynthesis in *Artemisia annua* seedlings [25,63]. Furthermore, the induction of artemisinin biosynthesis by *Penicillium oxalicum* B4 in *A. annua* was shown to be strongly dependent on the induced reactive oxygen species (ROS) production [63].

Biological activity against bacteria and fungi was also sought, following the “medicinal plant harbouring active fungal endophytes” model; this time spreading the screening across various species of *Artemisia*. Lu et al. [40] isolated and characterised three new metabolites, 6-isoprenylindole-3-carboxylic acid, 3b,5a-dihydroxy-6b-acetoxy-ergosta-7,22-diene, and 3b,5a-dihydroxy-6b-phenylacetyloxy-ergosta-7,22-diene, from the culture of the endophyte *Colletotrichum* sp. (host plant: *Artemisia annua)*. Various inhibitory activities were observed against *Candida albicans*, *Aspergillus niger*, and phytopathogenic fungi *Gaeumannomyces graminis* pvar. tritici, *Rhizoctonia cerealis*, and *Helminthosporium sativum*, as well as against the bacteria *Bacillus subtilis, Staphylococcus aureus*, *Micrococcus luteus*, and *Pseudomonas* sp. A strain of *Colletotrichum gloeosporioides* from *Artemisia mongolica* produces a novel secondary metabolite named colletotric acid inhibiting growth of *Bacillus subtilis*, *Staphylococcus aureus*, and *Micrococcus luteus* [64]. Three strains of *Aspergillus* spp. (i.e., SPS-02, SPS-04, and SPS-01) were isolated from *Artemisia annua* and were found to possess strong antimicrobial activities against the human pathogens *Escherichia coli*, *Staphylococcus aureus* and *Trichophyton rubrum*, and cytotoxic activities [41]. The authors also found an inhibitory effect on *Rhizoctonia cerealis* with a strain of *Mucor* sp. SPS-11, and the strongest antimicrobial activities observed against *Magnaporthe grisea* were exhibited by two strains of *Aspergillus fumigatus* (SPS-02) and *Cephalosporium* sp. (SPS-08). The same strain of *A. fumigatus* produced four ardeemin derivatives with various activities of reversing the multidrug-resistant phenotype in three cancer cell lines [65].

Seven endophytic fungal strains isolated from leaves of *Artemisia annua* were solvent-extracted and tested against the bacteria *Staphylococcus aureus*, *Streptococcus mutans*, *Salmonella enterica* serotype Typhi, and *Bacillus subtilis*, and the fungi *Malassezia furfur* and *Candida albicans* [66]. Three strains had the broadest range of antimicrobial activity (i.e., inhibition of all tested strains), one strain inhibited only *E. coli* and *S. aureus*, and three endophytes were active against three bacterial strains and *M. furfur*. None were active against *C. albicans*. Extracts obtained from cultures of endophytic fungi (i.e., *Rhodotorula* sp. and *Fusarium* sp.) isolated from *Artemisia argyi* proved cytotoxic against liver cancer cell lines (Hep G2) [67]. Similarly, *Pestalotiopsis hainanensis* was active against all tested cell lines (MCF-7 breast, COLO205 colon, and HL-60 leukemia), while *Phomopsis mali* and *Rhodotorula glutinis* inhibited the HL-60 leukemia cell line, *Alternaria* sp. was active only against the MCF-7 breast cell line, and *Colletotrichum gloeosporioides* inhibited growth of the COLO205 colon cell line [29].

(i) Five new fungal polyketides and (ii) two known analogues were isolated from EtOAc-extracted biomass of *Trichoderma koningiopsis* (strain QA-3): (i) *ent*-koninginin A, 1,6-di-*epi*-koninginin A, 15-hydroxykoninginin A, 10-deacetylkoningiopisinin D and koninginin T; and (ii) koninginin L and trichoketide A [68]. Both *ent*-koninginin A and trichoketide A inhibited *E. coli*, and the second compound also showed antifungal activity against *Bipolaris sorokiniana*, *Ceratobasidium cornigerum*, *Colletotrichum gloeosporioides*, *Fusarium graminearum*, *Fusarium oxysporum*, *Penicillium digitatum*, *Physalospora piricola* and *Valsa mali*. *Myrothecium rorideum* (strain IFB-E012) isolated from stems of *Artemisia annua* produced lumichrome, a compound with moderate cytotoxicity to the human cell line KB [51]. Furthermore, a new cytotoxic trichothecene macrolide dihydromyrothecine compound was isolated from the same strain [69] together with three new 10,13-cyclotrichothecane-based macrolides [70]. *Hypoxylon truncatum,* an endophytic fungus isolated from *Artemisia annua*, yielded two novel cytotoxic benzofluoranthene-based metabolites named daldinone C and daldinone D [42].

Previous studies exhibited antibiotic and antitumor activities provoked by secondary metabolites such as azaphilones, which were demonstrated to be of chemotaxonomic significance in the classification of the *Hypoxylon* species [71]. Antioxidant phenolic metabolites were also reported to be produced by fungal endophytes inhabiting *Artemisia capillaris*, *Artemisia indica* and *Artemisia lactiflora* [72].

Likewise, 12 endophytic fungi isolated from *Artemisia absinthium* were tested for their ability to infect grape berries and for their potential to reduce the infection with *Botrytis cinerea*. Three of the fungal strains reduced both the percentage of infected berries and lesion diameter [73]. Antagonism assays are the preliminary step in these kinds of studies. For instance, Myrchiang et al. [30] proposes as good biocontrol agents of *Phytophthora infestans* the use of *Trichoderma viride*, *Aspergillus fumigatus*, and *Penicillium atrovenetum*, isolated from *Artemisia nilagirica*. Liu et al. [52] found 14 fungal endophytes in *Artemisia annua* which produce antagonistic substances against four phytopathogenic fungi: *Fusarium graminearum, Rhizoctonia cerealis*, *Helminthosporium sativum*, and *Gerlachia nivalis*. Furthermore, inhibitory activities of culture broths were evaluated. Similarly, a series of endophytic fungi from seven species of *Artemisia* (i.e., *Artemisia tangutica*, *Artemisia brachyloba*, *Artemisia subulata*, *Artemisia argyi*, *Artemisia scoparia*, *Artemisia lavandulifolia*, and *Artemisia* sp.) were evaluated [23]. It is worth mentioning that both studies found that antagonism assays (i.e., dual culture) do not converge with toxicity assays (i.e., nutrient media amended).

Research on strains isolated from *A. annua* to pursue their degradation of triclosan, the emerging contaminant in the aqueous and soil environment, resulted in the identification of an active strain, B4, of *Penicillium oxalicum* [74]. Triclosan was removed from the culture media by fungal degradation with a rate of 97.26% after one hour of incubation in fungal culture medium (5 mg L^−1^). Effective results were displayed in nonsterile synthetic wastewater and completion of degradation of triclosan was obtained in two hours. It represents a pioneering study that demonstrated the degradation of the notorious antimicrobial pollutant triclosan.

During the last decade, limited studies have been conducted on bioprospection of the microbiome of *Artemisia* sp. Nevertheless, its endophytic fungal communities have been a source of 27 novel compounds and 22 already characterised molecules of which 35 were shown to have at least one interesting trait to be further pursued in biotechnology.

## 4. Conclusions

*Artemisia* is a large genus of perennial and annual plants with species producing a variety of interesting and active compounds. The early exploration of its endophytic communities indicates diversity with promise of further significant biological activity. Research of the endophytes associated with the genus has revealed both cosmopolitan and rare endophytic species with highly studied chemical profiles, active molecules, and developed in vitro culture techniques.

## Figures and Tables

**Table 1:** Endophytic fungi order, family, genus and host plants with values of relative abundance (RA) for genus, family, and order. Values are represented using a color scale with green representing the smallest value and red the highest value.

Order RA %	Family RA %	Genus	Genus RA % Per Host Plant/Plants											Host Plants
Amphisphaeriales 1.92	Pestalotiopsidaceae 2.13	*Pestalotiopsis*	0.9	1.19	11.76									A. capillaris* + A. indica* + A. lactiflora* (HK); *A. thuscula* (TF); A. argy* (CH)
Botryosphaeriales 7.69	Aplosporellaceae 2.13	*Aplosporella*	1.22											*A. thuscula* (TF)
	Botryosphaeriaceae 6.38	*Botryosphaeria*	41.67											*A. lavandulifolia* (CH)
		*Macrophomina*	2.44											*A. thuscula* (TF)
		*Neofusicoccum*	2.78	30.3	10.98									*A. lavandulifolia*; *A. thuscula* (LP); *A. thuscula* (TF)
Cantharellales 1.92	Ceratobasidiaceae 2.13	*Rhizoctonia*	16.67											*A. brachyloba* (CH)
Capnodiales 3.85	Cladosporiaceae 2.13	*Cladosporium*	2.44	2.77	5.88	8.69								*A. thuscula* (TF); A. annua* (CH); A. argy*; A. nilagirica* (IN)
	Dissoconiaceae 2.13	*Ramichloridium*	2.77											A. annua*
Diaporthales 3.85	Diaporthaceae 4.26	*Diaporthe*	5.56	16.67	8.33	9.09	5.88							*A. lavandulifolia*; *A. brachyloba*; *A. scoparia* (CH); *A. thuscula* (LP); A. argy*
		*Phomopsis*	6.3	5.88	36.36									*A. capillaris +*A. indica + A. lactiflora*; A. argy*; A. lactiflora* (CH)
Dothideales 1.92	Dothioraceae 2.13	*Aureobasidium*	0.9	3.66										*A. capillaris* + *A. indica* + A. lactiflora*; *A. thuscula* (TF)
Eurotiales 7.69	Trichocomaceae 8.51	*Aspergillus*	8.33	4.55	8.69									*A. lavandulifolia*; *A. thuscula* (LP); A. nilagirica*
		*Eupenicillium*	5.55											A. annua*
		*Paecilomyces*	2.77											A. annua*
		*Penicillium*	2.78	1.52	13.8	5.88	33.33	13.4						*A. lavandulifolia*; *A. thuscula* (LP); A. annua*; A. argy*; A. nilagirica*; *A. annua*
Glomerellales 1.92	Glomerellaceae 2.13	*Colletotrichum*	19.81	11.1	25	5.55	5.88	10.11	9.09					*A. capillaris* + *A. indica* + A. lactiflora*; *A. lavandulifolia*; *A. brachyloba*; A. annua*; A. argy*; *A. annua*; A. lactiflora* (CH)
Hypocreales 17.31	Clavicipitaceae 2.13	*Ephelis*	1.8											*A. capillaris* + *A. indica* + A. lactiflora*
	Hypocreaceae 4.26	*Hypocrea*	5.88	9.09										A. argy*; A. lactiflora*
		*Trichoderma*	4.34											A. nilagirica*
	Nectriaceae 4.26	*Fusarium*	5.55	5.88	4.34									A. annua*; A. argy*; A. nilagirica*
		*Nectria*	2.44											*A. thuscula* (TF)
	Stachybotryaceae 4.26	*Stachybotrys*	1.22											*A. thuscula* (TF)
		*Myrothecium*	N.A.											A. nilagirica*
		*Acremonium*	13											*A. annua*
		*Cephalosporium*	N.A.											*A. annua*
Mucorales 3.85	Mucoraceae4.26	*Rhizopus*	4.34											A. nilagirica*
		*Mucor*	N.A.											*A. annua*
Onygenales 1.92	Arthrodermataceae 2.13	*Arthroderma*	4.34											A. nilagirica*
Pleosporales 25.00	Sporormiaceae 2.13	*Preussia*	10.61	10.98										*A. thuscula* (LP); *A. thuscula* (TF)
	Didymosphaeriaceae 2.13	*Tremateia*	1.52											*A. thuscula* (LP)
	Leptosphaeriaceae 4.26	*Coniothyrium*	1.52											*A. thuscula* (LP)
		*Leptosphaeria*	N.A.											*A. annua*
	Pleosporaceae 14.89	*Alternaria*	24.30	13.89	75	25	66.67	33.33	33.33	23.5	36.36	28.79	41.46	*A. capillaris* + *A. indica* + A. lactiflora*; *A. lavandulifolia*; *A. tangutica*; *A. brachyloba*; *A. subulata*; *A. argy*; *A. scoparia*; A. argy*; A. lactiflora*; *A. thuscula* (LP); *A. thuscula* (TF)
		*Curvularia*	16.66	33.33	16.67	8.33	1.52							*A. tangutica* (CH); *A. brachyloba* (CH); *A. subulata* (CH); *A. argy*; *A. thuscula* (LP)
		*Drechslera*	0.9											A. capillaris + A. indica + A. lactiflora*
		*Edenia*	2.77											A. annua*
		*Neoplatysporoides*	3.03											*A. thuscula* (LP)
		*Stemphylium*	0.65	4.88										*A. thuscula* (LP); *A. thuscula* (TF)
		*Paraphoma*	1.52											*A. thuscula* (LP)
		*Phoma*	1.52	7.32	2.77	4.34								*A. thuscula* (LP); A. thuscula (TF); A. annua*; A. nilagirica*
Pleosporineae 1.92	Camarosporiaceae 2.13	*Camarosporium*	1.52	1.22										*A. thuscula* (LP); *A. thuscula* (TF)
Pythiales 1.92	Pythiaceae 2.13	*Pythium*	4.34											A. nilagirica*
Saccharomycetales 1.92	Debaryomycetaceae 2.13	*Meyerozyma*	9.09											A. lactiflora*
Sordariales 3.85	Chaetomiaceae 4.26	*Chaetomium*	2.44	11.76										*A. thuscula* (TF); A. argy*
		*Thielavia*	2.44											*A. thuscula* (TF)
Sporidiobolales 1.92	Sporidiobolaceae 2.13	*Rhodotorula*	5.88											A. argy*
Xylariales 9.62	Apiosporaceae 4.26	*Arthrinium*	5.88											A. argy*
		*Nigrospora*	5.56	33.33	75	25		3.03						*A. lavandulifolia*; *A. brachyloba*; *A. argy*; *A. scoparia*; *A. thuscula* (LP)
	Graphostromataceae 2.13	*Biscogniauxia*	2.44											*A. thuscula* (TF)
	Hypoxylaceae 2.13	*Hypoxylon*	N.A.											*A. annua*

RA % for genus—calculated as the number of isolates belonging to a genus divided by the total number of individuals. Each value of RA of a genus was calculated for a host (except *A. capillaris* + *A. indica* + *A. lactiflora*, where all three species were considered as one pool as the study did not allow other analysis). For the column RA %—genus, each cell represents a RA % value calculated for a host species or more; host species are shown in the right column, separated by “;”. Each cell corresponds to a host species in the right column. RA % for family and order—the number of times that one taxa was found in this survey divided by the total number of taxa (No. of families = 47, No. of order = 52); although no quantitative data was found for genera such as *Hypoxylon*, *Leptosphaeria*, *Mucor* and *Myrothecium*; these were used only to calculate the RA of the order/family. *—calculation of relative abundance made by the present authors using the data provided in the reviewed studies. For host plant species the geographical location was provided only at the first time of appearance in the text: CH = China, HK = Hong Kong island, IN = India, LP = La Palma island, TF = Tenerife island; except LP and TF to distinguish two locations for the same host species in the same study. N.A. – no available data (we used genera with no available data for RA % only if it provided a new family). Following studies were used for the table: *A. capillaris + A. indica* + A. lactiflora*—[22]; *A. thuscula*—[24]; A. argy*—[29]; A. lactiflora*—[39]; A. annua*—[25,40,41,42,43]; *A. tangutica*, *A. brachyloba*, *A. subulata*, *A. argy, A. lavandulifolia*—[23]; A. nilagirica*—[30].

**Table 2:** Literature review on bioactive endophytic fungi (EF) isolated from species of *Artemisia*: host species, plant part of isolation, fungal taxa, endophyte isolation method, bioactive compounds/extracts, class of compounds, bioactivity, and references.

*Artemisia* Species	Plant Part	EF Taxa	EF Identification Method	Compound/Extract	Class of Compounds	Bioactivity	Reference
*A. absinthium*	root	*Penicillium* spp. (strains B6C18 and B14C36)	N.A.	spore solution (10^6^ mL^−1^)	N.A.	Antifungal inhibition of diameter of lesions on vine berries: 75% and 91%	[73]
*A. annua*	stem	*Aspergillus* spp.	morphology	ethyl acetate crude extract	N.A.	Antibacterial, antifungal	[41]
		*Mucor* spp.					
		*Cephalosporium* spp.					
		*Fusarium* spp.				Antibacterial	
		*Aspergillus terreus* (strain IFB-E030)	N.A.	10-phenyl-[12]-cytochalasins Z17	Cytochalasan, alkaloids	Cytotoxic IC_50_ = 26.2 µM	[75]
		*Colletotrichum* sp.	morphology	3β-hydroxy-ergosta-5-ene	Ergosterol derivatives	Antifungal %I (200 µg mL^−1^): 77–85%	[40]
						Antimicrobial MIC (µg mL^−1^): 50–75	
				3-oxo-ergosta-4,6,8(11),22-tetraene	Triunsaturated steroids	Antifungal %I (200 µg mL^−1^): 75%	
						Antimicrobial MIC (µg mL^−1^): 25–75%	
				3β-hydroxy-5α,8α-epidioxy-ergosta-6,22-diene	Ergosterol peroxides	Antimicrobial MIC (µg mL^−1^): 50–75	
				6-isoprenylindole-3-carboxylic acid	indoles	Antifungal %I (200 µg mL^−1^): 57–77%	
						Antimicrobial MIC (µg mL^−1^): 25–75	
				3β,5α-dihydroxy-6β-acetoxy-ergosta-7,22-diene	sterols	Antifungal %I (200 µg mL^−1^): 75–77%	
						Antimicrobial MIC (µg mL^−1^): 50–100%	
				3β,5α-dihydroxy-6β-phenylacety- loxy-ergosta-7,22-diene		Antifungal %I (200 µg mL^−1^): 50–66%	
						Antimicrobial MIC (µg mL^−1^): 50–75	
		*Hypoxylon trunctatum*	morphology and 18S rDNA sequence	daldinone C	Benzo[j]fluoranthenes (polycyclic aromatic hydrocarbons)	Cytotoxic IC_50_ = 49.5 µM	[42]
				daldinone D		Cytotoxic IC_50_ = 41 µM	
		*Myrothecium roridum* (strain IFB-E012)		myrothecine A	Sesquiterpene-based trichothecenes	Cytotoxic IC_50_ = 8.5 µg mL^−1^	[69,76]
				myrothecine B		Cytotoxic IC_50_ = 0.76 µg mL^−1^	
				myrothecine C		Cytotoxic IC_50_ = 32.21 µg mL^−1^	
				roridin E	Macrocyclic trychothecenes	Cytotoxic IC_50_ = 0.03 µg mL^−1^	
				mytoxin B	Trychothecene macrolides	Cytotoxic IC_50_ = 0.002 µg mL^−1^	
				dihydromyrothecine C		Cytotoxic IC_50_ = 44.48 µM	
				roritoxin E		Cytotoxic IC_50_ = 0.26 µg mL^−1^, 10.54 µg mL^−1^	
		*Aspergillus fumigatus*		5-*N*-acetylardeemin	Pyrimidinones (aromatic heterocyclic diazenes)	Cytotoxic RF = 1.5–6 µM	[65]
				5-*N*-acetyl-15bβ-hydroxyardeemin		Cytotoxic RF = 2–9.5 µM	
				5-*N*-acetyl-15b-didehydroardeemin		Cytotoxic RF = 2.5–8 µM	
				5-*N*-acetyl-16α-hydroxyardeemin		Cytotoxic RF = 1.5–7 µM	
		*Colletotrichum gloeosporioides*	morphology	oligosaccharides extract elicitor for artemisinin	oligosaccharides	increment of artemisinin = 64.29%	[62]
		*Penicillium oxalicum*	ITS1, 5.8S, and ITS2	heat-killed mycelium degrading triclosan (TCS)	N.A.	max. adsorbing capacity of TCS = 17.60 mg g^−1^	[74]
	leaves	*Curvularia pallescens*		elicitor extract for artemisinin	N.A.	2–6% elicitor extract (*w/v*): artemisinin content = 1.21–3.47-times more than control	[59]
		*Cladosporium* sp.	morphology	ethyl acetate extract	N.A.	Antibacterial	[66]
	stem	*Paraphaeosphaeria nolinae* (strain IFB-E011)	N.A.	paranolin	polycyclic aromatic compounds (xanthene-based)	Cytotoxic IC_50_ values > 50 µg mL^−1^	[77]
		*Phomopsis* sp.		crude EtOAc extract = BB1, fraction Hex/EtOAc/MeOH = BB4, fractions MeOH = BB8, BB9, BB10	N.A.	Cytotoxic: IC_50_ (µg mL^−1^) BB1 = 17.11, BB4 = 0.7; %I (20 µg/mL) BB8 = 1.97, BB9 = 0.53, BB10 = 52.98	[78]
				tyrosol (purified from BB10)	Phenolic compounds		
*A. argyi*	N.A.	*Phomopsis* sp. (strain H31)	ITS1, 5.8S, and ITS2	ethyl acetate + ethyl acetate/water extract	N.A.	Antifungal %I (1 mg mL^−1^) = 34.57; Cytotoxic %I (20 µg mL^−1^) = 81.69	[29]
		*Cladosporium* sp. (strain H23)				Antifungal %I (1 mg mL^−1^) = 23.46	
		*Chaetomium* sp. (strain H40)				Antibacterial	
		*Penicillium* sp. (strain H9)				Antibacterial	
		*Pestalotiopsis* sp. (strain H14)				Antibacterial; Cytotoxic %I (20 µg mL^−1^) = 53.47, 71.43, 97.03	
		*Diaporthe* sp. (strain H26)				Antibacterial	
		*Trichoderma* sp. (strain H17)				Antibacterial	
		*Rhodotorula* sp. (strain H13)				Cytotoxic %I (20 µg mL^−1^) = 16.41, 23.56, 78.28	
		*Colletotrichum* sp. (strain H42)				Cytotoxic %I (20 µg mL^−1^) = 11.22, 26.95, 71.99	
	stem	*Nigrospora sphaerica*	morphology and ITS1, 5.8S, and ITS2	ethyl acetate crude extract	N.A.	Antifungal %I (1 mg mL^−1^): 48.37, 11.94, 24.92	[23]
		*Trichoderma koningiopsis* (strain QA-3)	ITS1, 5.8S, and ITS2	*ent*-koninginin A	Tricyclic polyketides	Antifungal MIC (µg mL^−1^): 8–64 Antibacterial MIC (µg mL^−1^): 8–64	[68]
				1,6-di-*epi*-koninginin A		Antifungal MIC (µg mL^−1^): 64 Antibacterial MIC (µg mL^−1^): 32–64	
				15-hydroxykoninginin A		Antifungal MIC (µg mL^−1^): 64 Antibacterial MIC (µg mL^−1^): 16–64	
				10-deacetylkoningiopisin	Bicyclic polyketides	Antifungal MIC (µg mL^−1^): 8–64 Antibacterial MIC (µg mL^−1^): 32–64	
				koninginin T	Tricyclic polyketides	Antifungal MIC (µg mL^−1^): 16 Antibacterial MIC (µg mL^−1^): 32–64	
				koninginin L		Antifungal MIC (µg mL^−1^): 16 Antibacterial MIC (µg mL^−1^): 32–64	
				trichoketide A	Bicyclic polyketides	Antifungal MIC (µg mL^−1^): 4–64 Antibacterial MIC (µg mL^−1^): 16–64	
*A. brachyloba*		*Alternaria alternata*	morphology and ITS1, 5.8S, and ITS2	ethyl acetate crude extract	N.A.	Antifungal %I (1 mg mL^−1^): 29.8, 33.2, 42.4	[23]
*A. capillaris*	flower	*Alternaria* sp. (strain Acap F1)	ITS1, 5.8S, and ITS2	ethyl acetate extract of mycelia; Trolox equivalent antioxidant content (TEAC), Total phenolic content (TPC)	phenolic acids, flavonoids	TEAC (µM trolox equivalent/100 mL culture) = 127.41; TPC (mg gallic acid equivalent/100 mL culture) = 13.71	[22,72]
		*Phomopsis* sp. (strain Acap. F3)				TEAC (µM trolox equivalent/100 mL culture) = 526.93; TPC (mg gallic acid equivalent/100 mL culture) = 49.22	
		*Phomopsis* sp. (strain AcapF4)				TEAC (µM trolox equivalent/100 mL culture) = 177.12; TPC (mg gallic acid equivalent/100 mL culture) = 18.43	
*A. indica*	leaves	*Colletotrichum* sp. (strain AiL1)				TEAC (µM trolox equivalent/100 mL culture) = 100.2; TPC (mg gallic acid equivalent/100 mL culture) = 6.88	
		*Xylaria* sp. (strain AiL3)				TEAC (µM trolox equivalent/100 mL culture) = 102.21; TPC (mg gallic acid equivalent/100 mL culture) = 8.69	
		*Diaporthe* sp. (strain AiL4)				TEAC (µM trolox equivalent/100 mL culture) = 217.76; TPC (mg gallic acid equivalent/100 mL culture) = 21.27	
*A. lactiflora*	N.A.	*Phomopsis* sp. (strain GYBH42)		ethyl acetate crude extract	N.A.	Cytotoxic, antioxidant	[39]
		*Alternaria* sp. (strain GYBH47)					
*A. lavandulifolia*	stem	*Cochliobolus geniculatus*	morphology and ITS1, 5.8S, and ITS2			Antifungal EC_50_ = 0.03; %I (1 mg mL^−1^) = 31.5 and 100	[23]
		*Botryosphaeria dothidea*				Antifungal %I (1 mg mL^−1^): 85.79, 22.4	
*A. mongolica*		*Colletotrichum gloeosporioides*	morphology	colletotric acid	Phenolic acids	Antibacterial MIC (µg mL^−1^): 25–50	[64]
*A. subulata*		*Curvularia spicifera*	morphology and ITS1, 5.8S, and ITS2	ethyl acetate crude extract	N.A.	Antifungal %I (1 mg mL^−1^): 8.95, 52.09, 77.9	[23]
*A. vulgaris*	N.A.	*Chalara* sp. (strain 6661)	N.A.	isofusidienol A, B, C, and D	chromones (benzopyran derivatives)	Antibacterial	[79]

EF—endophytic fungi; MIC—minimum inhibitory concentration; %I—percentage of inhibition, TEAC—trolox equivalent antioxidant capacity; TPC—total phenolic content; where both %I and EC_50_ are presented, it means that results were expressed as such depending on the pathogenic strains tested and conditions employed. InChI keys are provided as supplementary materials.

## Supplementary Materials

The following are available online at http://www.mdpi.com/2309-608X/4/2/53/s1.
